# FGF-23 is associated with increased risk of abdominal aortic calcification: A Mendelian randomization study with phosphate as a potential mediator

**DOI:** 10.1097/MD.0000000000050966

**Published:** 2026-10-02

**Authors:** Lei Song, Juan Chen, Cheng Xue, Chang Song, Jinhua Zhu, Daoliang Xu

**Affiliations:** a Division of Nephrology, People’s Hospital of Yangzhong City, Yangzhong, Jiangsu Province, China; b Medical College of Yangzhou University, Yangzhou, Jiangsu Province, China; c Kidney Institute, Division of Nephrology, Shanghai Changzheng Hospital, Second Affiliated Hospital of Naval Medical University, Shanghai, China; d Department of Cellular Biology and Anatomy, Medical College of Georgia at Augusta University, Augusta, GA; e Division of Nephrology, Subei People’s Hospital of Jiangsu Province, Medical College of Yangzhou University, Yangzhou, Jiangsu Province, China.

**Keywords:** abdominal aortic calcification, fibroblast growth factor 23, Mendelian randomization, phosphate

## Abstract

Fibroblast growth factor 23 (FGF23) is involved in the regulation of phosphate metabolism and has been linked to vascular calcification. However, the genetic association between FGF23 and abdominal aortic calcification (AAC) remains unclear. We conducted a bidirectional two-sample Mendelian randomization (MR) study to evaluate the association between genetically predicted circulating FGF23 levels and AAC. Univariable MR was performed to assess the primary association, followed by multivariable MR adjusting for serum calcium, 25-hydroxyvitamin D, parathyroid hormone, and chronic kidney disease. In addition, a two-step MR mediation analysis was used to assess whether phosphate might mediate this association. Univariable MR analysis suggested a modest association between genetically predicted circulating FGF23 levels and a higher risk of AAC (odds ratio = 1.082, 95% confidence interval = 1.016–1.151, *P* = .013). This association remained directionally consistent after separate adjustment for serum calcium, 25-hydroxyvitamin D, parathyroid hormone, and chronic kidney disease. In a secondary multivariable Mendelian randomization analysis using an alternative FGF23 genome-wide association study, the association was no longer statistically significant after adjustment for phosphate (odds ratio = 1.064, 95% confidence interval = 0.962–1.179, *P* = .224). Two-step MR mediation analysis suggested that serum phosphate may partially mediate the association between FGF23 and AAC, with a marginally significant indirect effect (inverse-variance weighted: β = 0.024, *P* = .049), accounting for approximately 30% of the total effect. Our findings provide suggestive evidence that genetically predicted higher FGF23 levels may be modestly associated with an increased risk of AAC, with serum phosphate potentially acting as a partial mediator.

## 1. Introduction

Fibroblast growth factor 23 (FGF23) is a hormone primarily secreted by osteocytes and osteoblasts, playing a critical role in regulating phosphate and vitamin D metabolism.^[1]^ It acts by inhibiting renal phosphate reabsorption and suppressing the production of 1,25-dihydroxyvitamin D, thus maintaining phosphate homeostasis.^[2]^ Elevated levels of FGF23 have been associated with several pathological conditions,^[3–5]^ including chronic kidney disease (CKD) and cardiovascular diseases.^[6]^

Abdominal aortic calcification (AAC) is a progressive condition characterized by calcium deposition in the abdominal aorta, which can lead to arterial stiffness, increased cardiovascular risk, and mortality.^[7]^ AAC is not only a marker of vascular aging but also a strong independent predictor of cardiovascular events and mortality.^[8,9]^ Estimates suggest that over 30% of individuals older than 60 years have detectable AAC. Studies in community-dwelling older adults have shown that AAC predicts cardiovascular mortality independently of established risk factors, highlighting its clinical significance beyond high-risk populations.^[9]^ Furthermore, AAC is commonly observed in patients with CKD and other disorders involving calcium-phosphate metabolism, suggesting a possible link between mineral dysregulation and vascular calcification. However, the high prevalence of AAC in the general older population indicates that its underlying mechanisms may extend beyond CKD-specific pathways.^[8–10]^

The relationship between FGF23 and vascular calcification, including AAC, has garnered increasing attention. Elevated FGF23 levels have been implicated in promoting vascular calcification through mechanisms that are not yet fully understood.^[11]^ It is hypothesized that disturbances in calcium and phosphate metabolism, common in CKD patients, may mediate this effect. However, despite numerous studies addressing these factors,^[12–14]^ there is a lack of direct genetic evidence supporting a causal relationship between FGF23 and AAC.

Most previous studies investigating the association between circulating FGF23 levels and AAC have been observational and, therefore, susceptible to residual confounding and reverse causality. Mendelian randomization (MR) uses genetic variants as instrumental variables (IVs) to assess the potential causal association between an exposure and an outcome, thereby reducing bias from conventional confounding and reverse causation. Because both elevated FGF23 levels and AAC can be observed not only in patients with CKD but also in broader populations, clarifying their relationship may have clinical relevance beyond the CKD setting. Therefore, we conducted a bidirectional two-sample MR study to evaluate the association between genetically predicted circulating FGF23 levels and AAC. We hypothesized that genetically predicted higher circulating FGF23 levels would be associated with an increased risk of AAC.

## 2. Materials and methods

### 2.1. Study design

We employed a two-sample MR approach to investigate the potential association between FGF23 and AAC. The study consisted of 3 stages. First, univariable MR analysis was performed to estimate the total association of FGF23 on AAC. Second, multivariable MR was conducted to adjust for potential confounders, including serum calcium, 25-hydroxyvitamin D, parathyroid hormone (PTH), and CKD. Third, a two-step MR mediation analysis was performed to evaluate whether serum phosphate may mediate the association between FGF23 and AAC (Fig. 1A).^[15,16]^ Valid MR inference relies on 3 core assumptions: the genetic instruments are associated with the exposure, are independent of exposure–outcome confounders, and affect the outcome only through the exposure.

**Figure 1.**
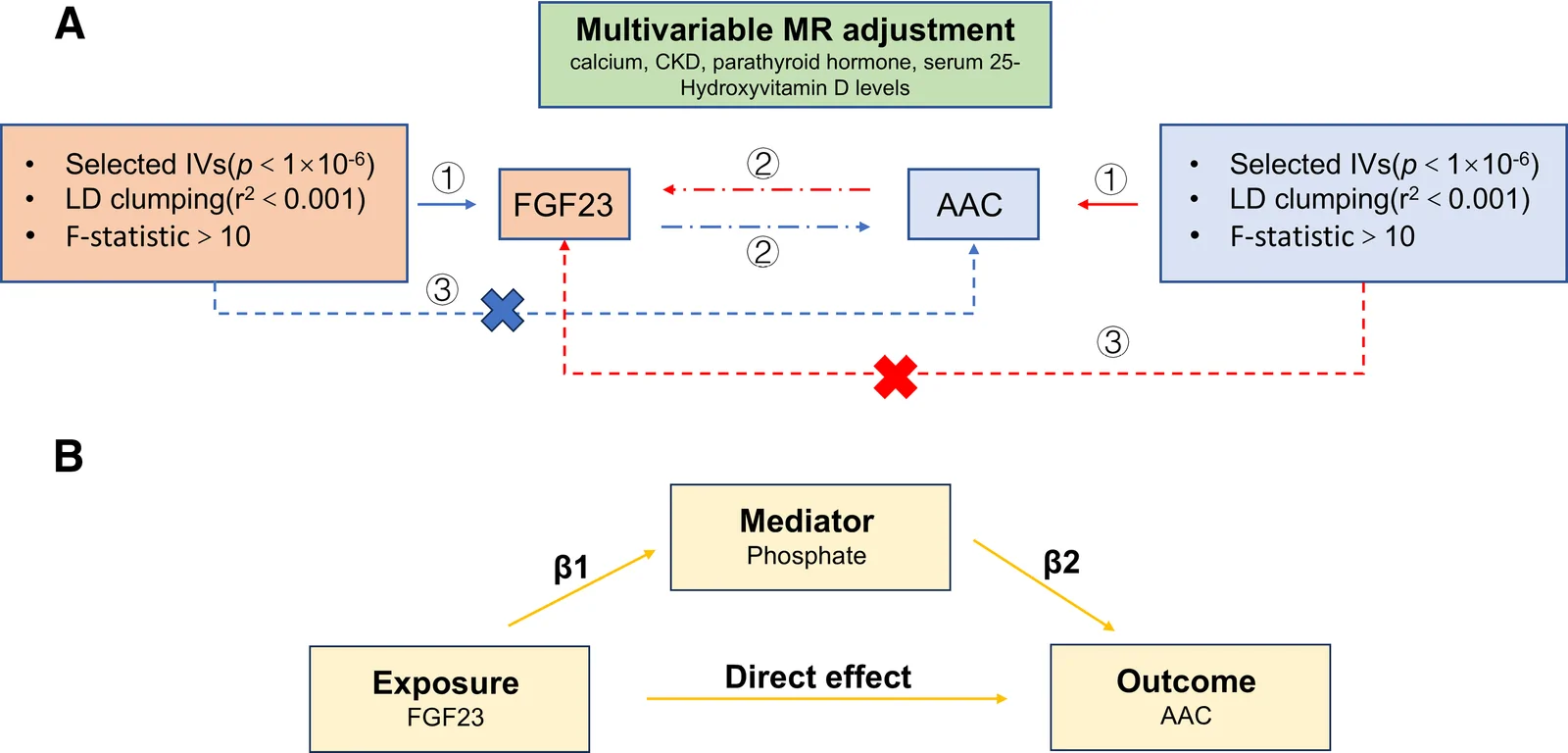
Description of the study design of univariable MR analysis, multivariable MR analysis, and mediation analysis. (A) ① The relevance assumption indicated that genetic instruments should be robustly associated with the exposure; ② a bidirectional MR analysis was used to explore the potential causal association between FGF23 and AAC; ③ the exclusion-restriction assumption indicated that the genetic instruments should only be associated with the outcome via the exposure without alternative pathways. Multivariable MR analysis was used to explore possible causal relationships between FGF23 and AAC when the confounders calcium, 25-hydroxyvitamin D, parathyroid hormone, and chronic kidney disease were used as multivariables separately. (B) The design of mediation analysis of the effect of FGF23 on AAC via phosphate. β1 represents the effect of FGF23 on the potential mediator, and β2 represents the effect of the mediator on AAC. The indirect effect was calculated as β1 × β2, and the direct effect was estimated as the total effect minus the indirect effect. AAC = abdominal aortic calcification, CKD = chronic kidney disease, FGF23 = fibroblast growth factor 23, IVs = instrumental variables, LD = linkage disequilibrium, MR = Mendelian randomization.

### 2.2. Data sources and instrument variables

Most datasets were derived from population cohorts in the Integrative Epidemiology Unit Genome-Wide Association Study (GWAS) database (https://opengwas.io/). The total sample size of FGF23 was 21,758 (id: ebi-a-GCST90012022); serum 25-hydroxyvitamin D was 443,734 (id: ebi-a-GCST010144); calcium levels were 357,831 (id: ebi-a-GCST90013999); PTH was 3301 (id: prot-a-2431); CKD included 216,743 participants (3902 cases and 212,841 controls; id: finn-b-N14_CHRONKIDNEYDIS). AAC summary statistics were obtained from the UK Biobank imaging sub-study (GCST90134614).^[17]^ AAC was quantified using a validated semiquantitative scoring system based on dual-energy X-ray absorptiometry lateral spine images.^[18]^ The score ranges from 0 to 24 and reflects the severity of calcification in the abdominal aorta. This phenotype has been widely used in epidemiological studies of vascular calcification and cardiovascular risk. The FGF23 GWAS was derived from the SCALLOP consortium, whereas the AAC GWAS was derived from the UK Biobank imaging sub-study. Therefore, substantial sample overlap between the primary exposure and outcome datasets is unlikely. The phosphate GWAS was also derived from UK Biobank; thus, potential sample overlap between the phosphate and AAC datasets cannot be completely excluded. Because only summary-level GWAS data were available, the exact number or proportion of overlapping participants could not be determined (Table 1).

**Table 1:** A detailed description of the GWAS data involved in this study.

GWAS trait	Population	Cases	Controls	Sample size	Number of SNPs	GWAS ID	Website
Fibroblast growth factor 23 levels	European	NA	NA	21,758	13,102,275	ebi-a-GCST90012022	https://gwas.mrcieu.ac.uk/datasets/ebi-a-GCST90012022/
Abdominal aortic calcification	European	NA	NA	31,786	9,572,557	GCST90134614	https://www.ebi.ac.uk/gwas/studies/GCST90134614
Serum 25-hydroxyvitamin D levels	European	NA	NA	443,734	15,847,859	ebi-a-GCST010144	https://gwas.mrcieu.ac.uk/datasets/ebi-a-GCST010144/
Calcium levels	European	NA	NA	357,831	10,783,676	ebi-a-GCST90013999	https://gwas.mrcieu.ac.uk/datasets/ebi-a-GCST90013999/
Phosphate	European	NA	NA	361,194	13,585,254	ukb-d-30810_irnt	https://gwas.mrcieu.ac.uk/datasets/ukb-d-30810_irnt/
Parathyroid hormone	European	NA	NA	3301	10,534,735	prot-a-2431	https://gwas.mrcieu.ac.uk/datasets/prot-a-2431/
Chronic kidney disease	European	3902	212,841	216,743	16,380,459	finn-b-N14_CHRONKIDNEYDIS	https://opengwas.io/datasets/finn-b-N14_CHRONKIDNEYDIS

GWAS = genome-wide association study, ID = identification, NA = not available, SNP = single-nucleotide polymorphism.

### 2.3. IVs selection and data harmonization

Single-nucleotide polymorphisms (SNPs) associated with FGF23 were selected using a significance threshold of *P* < 1 × 10^−6^ due to the limited number of genome-wide significant variants. Linkage disequilibrium (LD) clumping was performed (*r*^2^ < 0.001, 10 Mb window) to ensure independence. Instrument strength was assessed using the *F*-statistic, and SNPs with *F* > 10 were retained to minimize weak instrument bias.^[19]^ Instrument strength for each SNP was assessed using *F* = (β/SE)^2^, with *F* > 10 considered indicative of adequate instrument strength. Instruments with *F* > 10 were considered sufficiently strong to avoid weak instrument bias.^[19]^ In our study, all selected SNPs exhibited *F*-statistics ranging from 24 to 76, with a mean value of 51.3, indicating adequate instrument strength. Detailed SNP characteristics, including β, standard error (SE), and *F* values, are presented in Table S1, Supplemental Digital Content 1. To further account for potential weak instrument bias, we performed Mendelian Randomization using Robust Adjusted Profile Score (MR-RAPS), which provides consistent estimates under both weak instrument and balanced pleiotropy assumptions. A Steiger test was additionally conducted to confirm the causal direction between exposure and outcome traits by comparing the proportion of variance explained (*R*^2^) in the exposure versus outcome.

### 2.4. Bidirectional MR analysis

MR analysis was performed using 5 methods, including inverse-variance weighted (IVW), MR-Egger, weighted median, simple mode, and weighted mode.^[20]^ Heterogeneity was tested using Cochran’s *Q* statistic, and sensitivity analysis was conducted using the leave-one-out method to assess the reliability and feasibility of the data results.^[21]^ Pleiotropy was evaluated using the intercept from the MR-Egger method and the Mendelian Randomization Pleiotropy RESidual Sum and Outlier (MR-PRESSO) test.^[22]^ If the MR-PRESSO test indicated the presence of potential pleiotropy, the association was reassessed by eliminating outliers. The Steiger test was additionally conducted to confirm the causal direction between exposure and outcome by comparing the proportion of variance explained (*R*^2^) in the exposure versus outcome traits.

### 2.5. Multivariable MR analysis

Multivariable MR analysis was used to investigate the association of FGF23 with AAC, with the exclusion of confounding factors. FGF23 plays a role in regulating calcium and phosphorus metabolism^[23]^ and influences the progression of CKD^[24]^; therefore, serum calcium, 25-hydroxyvitamin D, PTH, and CKD were selected as possible confounders. When using the primary FGF23 GWAS dataset (ebi-a-GCST90012022), no eligible IVs remained after harmonization and clumping with the phosphate dataset. Therefore, we used the phosphate GWAS dataset (ID: met-a-364) and FGF23 (ID: prot-a-1093) for multivariable adjustment. For this secondary analysis, SNPs associated with FGF23 were selected using a relaxed significance threshold of *P* < 1 × 10^−6^, reflecting the limited number of genome-wide significant variants available. LD clumping was performed (*r*^2^ < 0.001, 10 Mb window) to ensure instrument independence. The instrument strength was adequate, with individual *F*-statistics > 10 for all variants.

### 2.6. Mediation analysis

A two-step approach was used to investigate potential mediating variables for the association of FGF23 on AAC. For the mediation analysis, we used the larger phosphate GWAS dataset (ID: ukb-d-30810_irnt). The met-a-364 dataset was selected for multivariable Mendelian randomization because it provided sufficient overlapping instruments with the alternative FGF23 GWAS, whereas the larger ukb-d-30810_irnt dataset was used for mediation analysis to maximize statistical power. We first examined the association of FGF23 with the mediating variable phosphate (β1) and then estimated the association of phosphate with the outcome AAC (β2). The indirect effect was calculated using the product-of-coefficients method by multiplying the effect of FGF23 on phosphate (β1) by the effect of phosphate on AAC (β2). The direct effect was estimated by subtracting the indirect effect from the total effect. The statistical significance of the indirect effect was assessed using the delta method. The mediating effect of MR design is shown in Figure 1B. This two-step MR mediation approach relies on several key assumptions^[25]^: the genetic instruments for the exposure (FGF23) must be associated with the exposure but not with the mediator or outcome through other pathways (relevance and exclusion restriction), there should be no confounding between the mediator (phosphate) and the outcome (AAC), and there should be no interaction between the exposure and the mediator in their effect on the outcome. These assumptions ensure that the mediation effect is not biased by horizontal pleiotropy or collider bias. To further minimize bias from horizontal pleiotropy, we also examined pleiotropy using MR-Egger and MR-PRESSO in both steps of the mediation analysis.

MR analyses were conducted using R software (version 4.3.2; R Foundation for Statistical Computing), TwoSampleMR (version 0.6.1; MRC Integrative Epidemiology Unit, University of Bristol, https://github.com/MRCIEU/TwoSampleMR), the MR-PRESSO package (version 1.0.0; Ron Do Lab, Icahn School of Medicine, https://github.com/rondolab/MR-PRESSO), and the ieugwasr package (version 1.0.0; MRC Integrative Epidemiology Unit, University of Bristol, https://github.com/MRCIEU/ieugwasr), with a two-sided *P*-value < .05 considered significant. All estimates were expressed as odds ratios (ORs). Statistical power for the primary MR analysis was estimated using the mRnd calculator (https://shiny.cnsgenomics.com/mRnd/), based on the observed effect size, the total variance explained by the genetic instruments, and a two-sided significance level of 0.05. This study used publicly available summary-level GWAS data and involved no new collection of individual-level data; therefore, additional institutional ethical approval and informed consent were not required.

## 3. Results

### 3.1. Bidirectional MR analysis

Univariable MR analysis showed that genetically predicted FGF23 levels were associated with an increased risk of AAC (IVW OR = 1.082, 95% confidence interval [CI] = 1.016–1.151, *P* = .013; Fig. 2A and Table 2). We estimated the statistical power assuming the observed effect size (OR = 1.082) and total variance explained (Σ*R*^2^ = 0.016). The analysis had approximately 79% power to detect the observed effect size at α = 0.05. Table S1, Supplemental Digital Content 1 lists information on 13 SNPs associated with circulating FGF23 levels. No horizontal pleiotropy (MR-Egger regression analysis: *P* = .439; MR-PRESSO: *P* = .878), heterogeneity (IVW: *P* = .892; MR-Egger: *P* = .887), or outliers were found in Table 3. Figure 2B presents the forest plot of MR estimates showing the causal effect of genetically predicted FGF23 levels on AAC. The scatter plot demonstrated the estimates of the association of SNP on AAC under the different methods (Fig. 2C). The funnel plot shows that the 13 SNPs are symmetrically distributed on both sides of the total IVW effect (Fig. 2D). Leave-one-out analysis showed that AAC was not significantly affected by a single SNP (Fig. 2E). The Steiger test found no directional errors and did not exclude any SNPs. The Steiger directionality test supported a directionality from FGF23 to AAC (*P* < .001). The MR-RAPS method yielded a consistent causal estimate (β = 0.080, SE = 0.033, *P* = .017; Fig. 2F). The MR-PRESSO global test indicated no evidence of horizontal pleiotropy.

**Table 2:** Bidirectional Mendelian randomization analysis between fibroblast growth factor 23 and abdominal aortic calcification.

Exposure	Outcome	Method	Number of SNPs	β	*P* value	OR (95% CI)
Fibroblast growth factor 23 levels	Abdominal aortic calcification	MR-Egger	13	0.001	.990	1.001 (0.821–1.221)
		Weighted median	13	**0.093**	**.029**	**1.097 (1.010–1.192**)
		IVW	13	**0.079**	**.013**	**1.082 (1.016–1.151**)
		Simple mode	13	0.100	.211	1.105 (0.953–1.281)
		Weighted mode	13	0.101	.168	1.106 (0.967–1.266)
Abdominal aortic calcification	Fibroblast growth factor 23 levels	MR-Egger	6	−0.014	.974	0.986 (0.452–2.152)
		Weighted median	6	0.018	.901	1.018 (0.769–1.348)
		IVW	6	−0.008	.944	0.992 (0.788–1.249)
		Simple mode	6	−0.023	.921	0.978 (0.641–1.492)
		Weighted mode	6	−0.002	.992	0.998 (0.689–1.445)

Bold values indicate statistically significant positive results (*P* < 0.05).

CI = confidence interval, IVW = inverse-variance weighted, MR = Mendelian randomization, OR = odds ratio, β = effect size.

**Table 3:** Sensitivity analysis of fibroblast growth factor 23 on abdominal aortic calcification.

Exposure	Outcome	Heterogeneity test				Pleiotropy test			
		IVW		MR-Egger		MR-Egger regression		MR-PRESSO	
		*Q*	*P* value	*Q*	*P* value	Intercept	*P* value	Outliers number	*P* value
Fibroblast growth factor 23 levels	Abdominal aortic calcification	6.439	.892	5.794	.887	0.007	.439	NA	.878
Abdominal aortic calcification	Fibroblast growth factor 23 levels	5.898	.316	5.897	.207	0.0003	.989	NA	.384

IVW = inverse-variance weighted, MR = Mendelian randomization, MR-PRESSO = MR Pleiotropy RESidual Sum and Outlier, NA = not available, *Q* = Cochran’s *Q* statistic.

**Figure 2.**
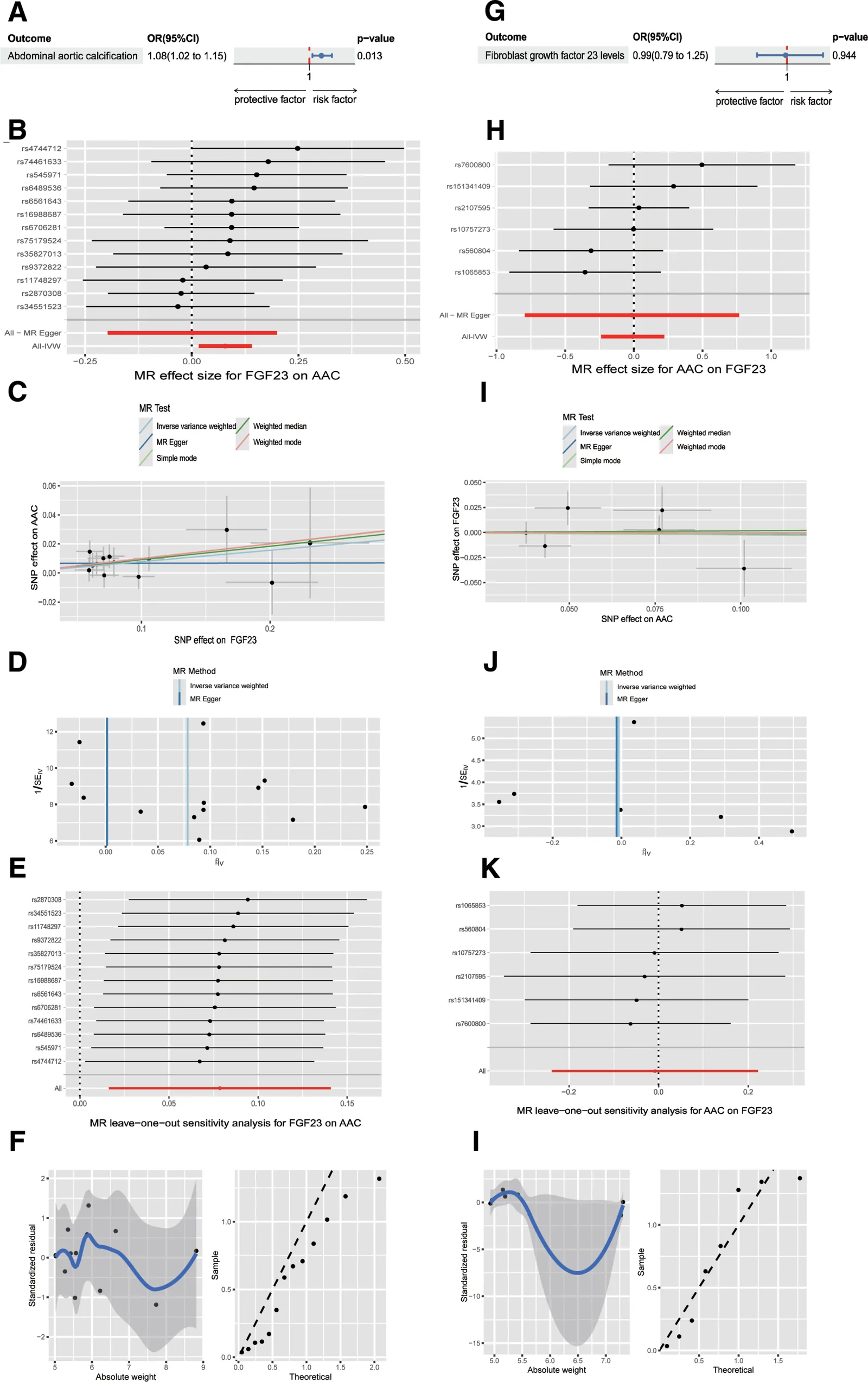
Primary and reverse MR analyses between FGF23 and AAC. (A) Forest plot showing the estimated association of FGF23 on AAC. (B) Forest plot of MR estimates for the effect of genetically predicted circulating FGF23 levels on AAC. (C) Scatterplot demonstrating the distribution of the association of FGF23 on AAC. (D) Funnel plot of MR estimates for the effect of circulating FGF23 on AAC. (E) Leave-one-out plot analysis demonstrating the robustness of the association of FGF23 on AAC. (F) MR-RAPS outlier-robust scatter plot for the effect of genetically predicted FGF23 on AAC. (G) Forest plot showing the estimated association of AAC on FGF23. (H) Forest plot of MR estimates for the effect of genetically predicted AAC on FGF23. (I) Scatterplot demonstrating the distribution of the association of AAC on FGF23. (J) Funnel plot of MR estimates for the effect of AAC on FGF23. (K) Leave-one-out plot analysis demonstrating the robustness of the association of AAC on FGF23. (L) MR-RAPS outlier-robust scatter plot for the effect of genetically predicted AAC on FGF23. AAC = abdominal aortic calcification, CI = confidence interval, FGF23 = fibroblast growth factor 23, IVW = inverse-variance weighted, MR = Mendelian randomization, MR-RAPS = Mendelian Randomization Robust Adjusted Profile Score, OR = odds ratio, SNP = single-nucleotide polymorphism.

Reverse MR analysis showed no evidence of an association of AAC on FGF23 levels (OR = 0.992, 95% CI = 0.788–1.249, *P* = .944; Fig. 2G and Table 2). Table S2, Supplemental Digital Content 2 lists information on 6 SNPs used for reverse MR. No horizontal pleiotropy (MR-Egger regression analysis: *P* = .989; MR-PRESSO: *P* = .384), heterogeneity (IVW: *P* = .316; MR-Egger: *P* = .207), or outliers were found in Table 3. Figure 2H presents the forest plot of MR estimates showing the causal effect of genetically predicted AAC on FGF23 levels. The scatter plot demonstrated the estimates of the association of SNP on AAC under the different methods (Fig. 2I). The funnel plot shows that the 6 SNPs are symmetrically distributed on both sides of the total IVW effect (Fig. 2J). Leave-one-out analysis showed that AAC was not significantly affected by a single SNP (Fig. 2K). The MR-RAPS method yielded a consistent causal estimate (β = 0.030, SE = 0.100, *P* = .762; Fig. 2L).

### 3.2. Multivariable MR analysis

As shown in Table 4 and Figure 3, the association between FGF23 and AAC persisted after adjustment for serum calcium, 25-hydroxyvitamin D, PTH, and CKD, which are potential confounders (calcium levels, IVW: OR = 1.097, 95% CI = 1.023–1.177, *P* = .009; serum 25-hydroxyvitamin D levels, IVW: OR = 1.091, 95% CI = 1.028–1.157, *P* = .004; PTH, IVW: OR = 1.073, 95% CI = 1.000–1.151, *P* = .049; CKD, IVW: OR = 1.096, 95% CI = 1.008–1.191, *P* = .032). Specifically, we used the phosphate GWAS dataset (ID: met-a-364) and FGF23 (ID: prot-a-1093) in our multivariable analysis, ultimately identifying 4 SNPs: rs10089499, rs1466522, rs2399393, rs7220979 after harmonization and LD clumping. FGF23 levels were associated with an increased risk of AAC (IVW: OR = 1.048, 95% CI = 1.014–1.083, *P* = .005). However, this association was no longer statistically significant after adjustment (IVW: OR = 1.064, 95% CI = 0.962–1.179, *P* = .224).

**Table 4:** MVMR results of fibroblast growth factor 23 on the risk of abdominal aortic calcification.

Exposure	Outcome	Adjustment	Beta	OR (95% CI)	*P* value
Fibroblast growth factor 23 levels (ID: ebi-a-GCST90012022)	Abdominal aortic calcification	Calcium levels	**0.093**	**1.097 (1.023–1.177**)	**.009**
		Serum 25-hydroxyvitamin D levels	**0.087**	**1.091 (1.028–1.157**)	**.004**
		Parathyroid hormone	**0.070**	**1.073 (1.000–1.151**)	**.049**
		Chronic kidney disease	**0.091**	**1.096 (1.008–1.190**)	**.032**
Fibroblast growth factor 23 levels (ID: prot-a-1093)	Abdominal aortic calcification	Phosphate (ID: met-a-364)	**0.069**	1.064 (0.962–1.179)	.224

The phosphate-adjusted MVMR was conducted as a secondary analysis using an alternative FGF23 GWAS dataset (prot-a-1093) and is therefore distinct from, and not directly comparable with, the primary MVMR analyses based on ebi-a-GCST90012022.

Bold values indicate statistically significant positive results (*P* < 0.05).

CI = confidence interval, GWAS = genome-wide association study, MVMR = multivariable Mendelian randomization, OR = odds ratio.

**Figure 3.**
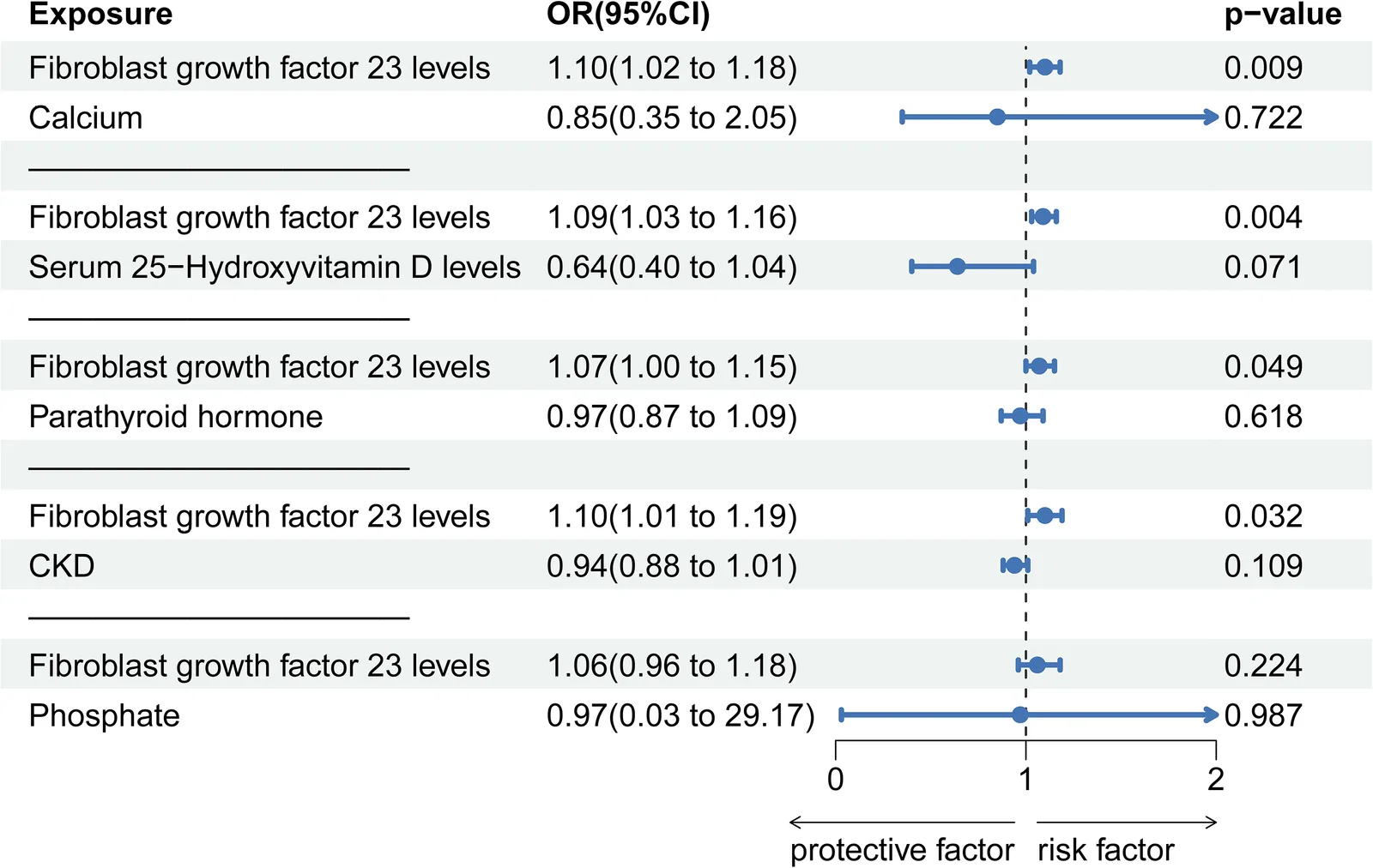
MVMR analyses of the association between FGF23 and AAC after adjustment for selected covariates. This forest plot shows the effect of FGF23 on AAC after correction for different potential confounders separately. Each estimation point in the plot is represented by a solid circle, the size of which is proportional to the inverse of the standard error. The horizontal line next to the solid circle represents the 95% CI. A reference line (OR of 1) was included for comparison, with values below 1 indicating protective factors and values above 1 indicating risk factors. Adjustment variables included calcium, 25-hydroxyvitamin D, parathyroid hormone, chronic kidney disease, and phosphate, with an outcome of AAC. The phosphate-adjusted MVMR was conducted as a secondary analysis using an alternative FGF23 GWAS dataset (prot-a-1093) and is therefore distinct from and not directly comparable with the primary MVMR analyses based on ebi-a-GCST90012022. AAC = abdominal aortic calcification, CI = confidence interval, CKD = chronic kidney disease, FGF23 = fibroblast growth factor 23, GWAS = genome-wide association study, MVMR = multivariable Mendelian randomization, OR = odds ratio.

### 3.3. Mediation analysis

To evaluate whether serum phosphate may lie on the pathway linking genetically predicted FGF23 to AAC, we implemented a two-step MR framework to quantify the indirect (phosphate-mediated) and direct (non-mediated) effects of FGF23 on AAC. After harmonization and LD clumping, 8 independent FGF23-associated SNPs and 157 phosphate-associated SNPs were retained for the first and second steps, respectively; detailed information is shown in Table S3, Supplemental Digital Content 3 and Table S4, Supplemental Digital Content 4. The two-step MR analysis yielded a marginally significant indirect effect consistent with possible partial mediation by phosphate (β = 0.024, 95% CI = 0.001–0.046, *P* = .049), accounting for approximately 30.4% of the total effect (Table 5). Specifically, the effect of FGF23 on phosphate accounted for the first-order effect (β1 = 0.198, *P* = .016), while the second-order effect was the influence of phosphate on AAC (β2 = 0.119, *P* < .001); the direct effect was β = 0.055.

**Table 5:** Mediation effect of fibroblast growth factor 23 on abdominal aortic calcification through phosphate.

Mediator	Total effect		Effect step 1 (FGF23 on phosphate)			Effect step 2 (phosphate on AAC)			Direct effect	Indirect effect		Mediated proportion
	β	*P* value	β1	*P* value	OR (95% CI)	β2	*P* value	OR (95% CI)	β	β (95% CI)	*P* value	
Phosphate	**0.079**	**.013**	**0.198**	**.016**	**1.219 (1.038–1.432**)	**0.119**	**<.001**	**1.127 (1.063–1.194**)	**0.055**	**0.024 (0.001–0.046**)	**.049**	**30.4**%

Bold values indicate statistically significant positive results (*P* < 0.05).

AAC = abdominal aortic calcification, CI = confidence interval, FGF23 = fibroblast growth factor 23, OR = odds ratio, β = effect size, β1 = effect size for FGF23 on phosphate, β2 = effect size for phosphate on AAC.

## 4. Discussion

In this study, we identified a genetic association between elevated FGF23 levels and AAC using a two-sample MR approach, with findings consistent with a potential causal association. Univariable MR analysis showed that genetically predicted FGF23 levels were associated with a higher risk of AAC, and this association persisted after adjustment for serum calcium, 25-hydroxyvitamin D, PTH, and CKD. In addition, the two-step MR mediation analysis suggested that phosphate may partially mediate the association between FGF23 and AAC, although this mediation effect was modest and marginally significant. The observed effect size was also modest, and therefore the magnitude of the association should not be overinterpreted. In the context of MR, such estimates reflect lifelong genetically predicted differences in exposure rather than acute changes in circulating FGF23 levels. Thus, even a small genetic association may still be biologically relevant, particularly for multifactorial conditions such as vascular calcification. Reverse MR analysis showed no evidence that genetically predicted AAC was associated with circulating FGF23 levels. The Steiger directionality test further supported the direction from FGF23 to AAC (*P* < .001). In addition, reverse MR estimates were nonsignificant across several methods, including MR-Egger, weighted median, IVW, and MR-RAPS, providing additional support for the directionality of the observed association. Overall, these findings support the biological and epidemiological plausibility of a possible role of FGF23 in AAC and warrant further translational research.

Although elevated circulating FGF23 has been associated with an increased risk of vascular calcification and cardiovascular events, most prior studies were observational, therefore prone to residual confounding and reverse causality. To date, few studies have explored the causal relationship between FGF23 and AAC using genetic approaches. Previous observational studies have suggested that FGF23 is involved in vascular calcification,^[26–28]^ particularly in patients with CKD.^[29]^ Elevated FGF23 levels have been associated with increased cardiovascular mortality and arterial calcification in CKD patients.^[30]^ Our study differs from these previous works by providing genetic evidence suggestive of a contributory role of FGF23 in AAC. In addition, our results align with findings from studies indicating that phosphate metabolism may play a role in vascular calcification. However, the precise mechanisms linking FGF23 to vascular calcification remain unclear in many of these studies,^[28,31,32]^ whereas our two-step MR analysis suggests that phosphate may potentially contribute to the observed association as a partial mediator, though the indirect effect is modest.

Our genetic evidence linking FGF23 to AAC, potentially through phosphate, is consistent with prior experimental studies that elucidate the mechanistic underpinnings of vascular calcification. Elevated phosphate has been shown to induce vascular smooth muscle cell osteogenic differentiation, with upregulation of bone-related markers such as Runx2 and osteocalcin.^[14]^ Moreover, FGF23 may enhance this process, especially under hyperphosphatemic conditions, suggesting a synergistic role in vascular calcification.^[11]^ Phosphate not only stimulates vascular smooth muscle cell transdifferentiation but also promotes matrix vesicle release and vascular smooth muscle cell apoptosis, both of which act as nucleation sites for hydroxyapatite deposition.^[13,33]^ At the molecular level, the interaction between FGF23 and soluble Klotho has emerged as a critical determinant of phosphate homeostasis and vascular health. Wungu et al provided mechanistic insight into how Klotho modulates FGF23 signaling in aortic valve tissues under CKD conditions, promoting pro-calcific changes.^[28]^ These biological pathways provide a plausible mechanistic rationale supporting the potential mediation role of phosphate, reinforcing the plausibility and consistency of our MR findings in the context of chronic phosphate imbalance, such as in CKD.

The results of this study can be explained by FGF23’s role in regulating phosphate homeostasis. FGF23 plays a central role in mineral metabolism by directly downregulating sodium-phosphate co-transporters (NaPi-IIa and NaPi-IIc) in the renal proximal tubules, thereby promoting urinary phosphate excretion.^[34]^ In parallel, FGF23 suppresses the renal expression of 1α-hydroxylase, reducing the synthesis of 1,25-dihydroxyvitamin D and thereby limiting intestinal calcium and phosphate absorption. These regulatory effects are mediated through its interaction with the Klotho-FGFR1 complex in renal and vascular tissues. In CKD, persistent elevation of FGF23 may contribute to dysregulation in phosphate homeostasis.^[2,35]^ In CKD, persistent elevations in FGF23 reflect compensatory responses to phosphate retention. Although FGF23 physiologically promotes phosphate excretion, impaired kidney function limits its effectiveness, leading to sustained disturbances in phosphate homeostasis.^[13,36]^ The accumulation of phosphate in vascular tissues can enhance calcium deposition,^[33,37]^ contributing to the progression of AAC. Therefore, our findings suggest that FGF23 may be associated with AAC partly through phosphate metabolism, which is consistent with the established role of phosphate in vascular calcification.

Beyond genetic associations, growing evidence from clinical cohorts has established the prognostic relevance of vascular calcification biomarkers in high-risk populations. For instance, Jia et al demonstrated that serum osteocalcin was independently associated with AAC in hemodialysis patients, highlighting the role of bone-derived hormones in vascular pathology.^[38]^ Similarly, Milovanova et al reported that reduced α-Klotho levels were associated with increased cardiovascular and all-cause mortality in patients with stage 3b–4 CKD, underscoring the systemic effects of disrupted mineral metabolism.^[39]^ Martín-Carro et al further supported this notion by identifying soluble α-Klotho as a potential circulating biomarker for both renal and vascular impairment.^[40]^ These findings align with our MR results and suggest that phosphate-related hormonal axes may contribute to the pathogenesis and prognosis of AAC.

Our study has several limitations. First, mediation MR assumes no unmeasured confounding between the mediator and outcome, which cannot be entirely verified using summary-level data. Future studies with larger multi-ancestry GWAS and individual-level replication are warranted.^[41,42]^ Second, because both the phosphate and AAC GWAS datasets were derived from UK Biobank-related resources, potential sample overlap between the mediator and outcome datasets could not be fully excluded. This may introduce bias into the mediation estimate. Therefore, the phosphate-mediated effect should be interpreted cautiously as suggestive rather than definitive evidence of mediation. Third, the GWAS datasets used in this study were primarily derived from individuals of European ancestry, which may limit the generalizability of our findings to other populations. Future work should utilize multi-ancestry GWAS and validate findings in independent cohorts with individual-level AAC imaging and phosphate measurements. In addition, the estimated statistical power of the primary MR analysis was approximately 79%, slightly below the conventional 80% threshold. Although the primary IVW analysis yielded a statistically significant association, this modest power may increase the risk of Type II error, particularly for secondary and mediation analyses with smaller effect sizes. Therefore, larger GWAS datasets are needed to validate these findings.

In conclusion, our findings provide genetic evidence suggestive of a potential contributory role of FGF23 in AAC, with phosphate possibly acting as a partial mediator. These findings provide preliminary evidence suggestive of a role for the FGF23–phosphate axis in vascular calcification pathophysiology. However, given the modest effect size and the marginally significant mediation effect, these results should be considered hypothesis-generating rather than clinically actionable. Any potential clinical relevance – such as the utility of FGF23 or phosphate as therapeutic targets or biomarkers for AAC risk – would require substantial validation in larger, multi-ancestry cohorts and ideally in randomized controlled trials before firm conclusions can be drawn.

## Author contributions

**Conceptualization:** Lei Song, Juan Chen, Cheng Xue, Chang Song.

**Data curation:** Lei Song, Juan Chen, Cheng Xue, Jinhua Zhu.

**Formal analysis:** Lei Song, Juan Chen.

**Software:** Juan Chen, Cheng Xue, Chang Song, Jinhua Zhu.

**Validation:** Juan Chen, Cheng Xue, Chang Song.

**Supervision:** Cheng Xue, Daoliang Xu.

**Investigation:** Chang Song.

**Resources:** Chang Song, Daoliang Xu.

**Project administration:** Jinhua Zhu, Daoliang Xu.

**Funding acquisition:** Daoliang Xu.

**Writing – original draft:** Lei Song, Juan Chen, Cheng Xue, Jinhua Zhu.

**Writing – review & editing:** Lei Song, Juan Chen, Cheng Xue.








