# Supplementary material for: FGF-23 is associated with increased risk of abdominal aortic calcification: A Mendelian randomization study with phosphate as a potential mediator
Source: Medicine (Baltimore). 2026 Oct 2;105(40):e50966. doi: 10.1097/MD.0000000000050966 (PMC13633000; doi:10.1097/MD.0000000000050966)
Supplement: Supplementary file 1 [file medi-105-e50966-s001.docx]

**Supplementary table 1.** 13 SNPs of FGF23 used as genetic tools for MR analysis.

| Exposure | Outcome | SNP | Effect allele | Other allele | β | Se | P | F- statistic |
| --- | --- | --- | --- | --- | --- | --- | --- | --- |
| FGF23 | AAC | rs35827013 | G | A | -0.062 | 0.012 | 6.33403e-07 | 24.9182745759355 |
|  |  | rs6706281 | A | C | -0.106 | 0.012 | 2.42885e-18 | 76.3061028015047 |
|  |  | rs11748297 | A | G | -0.071 | 0.011 | 3.565e-10 | 39.36548647 |
|  |  | rs34551523 | A | G | 0.202 | 0.036 | 1.81702e-08 | 31.6784619052626 |
|  |  | rs9372822 | C | T | 0.059 | 0.011 | 1.167e-07 | 27.8431877397957 |
|  |  | rs74461633 | T | C | 0.166 | 0.032 | 1.54301e-07 | 27.5198468902454 |
|  |  | rs4744712 | C | A | 0.060 | 0.010 | 1.08101e-08 | 32.8402065675633 |
|  |  | rs545971 | T | C | 0.075 | 0.012 | 6.7112e-11 | 42.5311264604932 |
|  |  | rs6489536 | C | G | 0.070 | 0.012 | 7.77195e-09 | 33.1081262211511 |
|  |  | rs6561643 | T | A | 0.065 | 0.012 | 3.17797e-08 | 30.435329076476 |
|  |  | rs2870308 | C | A | 0.098 | 0.013 | 4.70111e-15 | 61.0870483037044 |
|  |  | rs16988687 | C | T | 0.079 | 0.015 | 7.44303e-08 | 28.9077068039639 |
|  |  | rs75179524 | C | T | 0.231 | 0.046 | 5.95703e-07 | 24.9341011406491 |
