# Supplementary material for: FGF-23 is associated with increased risk of abdominal aortic calcification: A Mendelian randomization study with phosphate as a potential mediator
Source: Medicine (Baltimore). 2026 Oct 2;105(40):e50966. doi: 10.1097/MD.0000000000050966 (PMC13633000; doi:10.1097/MD.0000000000050966)
Supplement: Supplementary file 2 [file medi-105-e50966-s002.docx]

**Supplementary table 2.** 6 SNPs of AAC used as genetic tools for MR analysis.

| Exposure | Outcome | SNP | Effect allele | Other allele | β | Se | P | F- statistic |
| --- | --- | --- | --- | --- | --- | --- | --- | --- |
| AAC | FGF23 | rs560804 | T | C | -0.043 | 0.008 | 1.5e-08 | 32.01901448 |
|  |  | rs1065853 | T | G | -0.101 | 0.014 | 3.1e-13 | 53.16059144 |
|  |  | rs7600800 | C | A | 0.050 | 0.010 | 2.9e-07 | 26.3071241 |
|  |  | rs151341409 | C | A | -0.077 | 0.014 | 6.4e-08 | 29.2306591 |
|  |  | rs2107595 | A | G | 0.076 | 0.010 | 2.7e-13 | 53.39557839 |
|  |  | rs10757273 | A | C | 0.037 | 0.008 | 8.2e-07 | 24.31629084 |
