# Supplementary material for: FGF-23 is associated with increased risk of abdominal aortic calcification: A Mendelian randomization study with phosphate as a potential mediator
Source: Medicine (Baltimore). 2026 Oct 2;105(40):e50966. doi: 10.1097/MD.0000000000050966 (PMC13633000; doi:10.1097/MD.0000000000050966)
Supplement: Supplementary file 3 [file medi-105-e50966-s003.docx]

**Supplementary Table 3**. 8 SNPs of FGF23 used as genetic tools for two-step analysis.

| Exposure | Outcome | SNP | Effect allele | Other allele | β | Se | P | F- statistic |
| --- | --- | --- | --- | --- | --- | --- | --- | --- |
| FGF23 | Phosphate | rs11748297 | A | G | -0.071 | 0.011 | 3.57E-10 | 39.37 |
|  |  | rs2870308 | C | A | 0.098 | 0.013 | 4.70E-15 | 61.09 |
|  |  | rs34551523 | A | G | 0.202 | 0.036 | 1.82E-08 | 31.68 |
|  |  | rs4744712 | C | A | 0.060 | 0.010 | 1.08E-08 | 32.84 |
|  |  | rs545971 | T | C | 0.075 | 0.012 | 6.71E-11 | 42.53 |
|  |  | rs6489536 | C | G | 0.070 | 0.012 | 7.77E-09 | 33.11 |
|  |  | rs6561643 | T | A | 0.065 | 0.012 | 3.18E-08 | 30.44 |
|  |  | rs6706281 | A | C | -0.106 | 0.012 | 2.43E-18 | 76.31 |
