# Supplementary material for: FGF-23 is associated with increased risk of abdominal aortic calcification: A Mendelian randomization study with phosphate as a potential mediator
Source: Medicine (Baltimore). 2026 Oct 2;105(40):e50966. doi: 10.1097/MD.0000000000050966 (PMC13633000; doi:10.1097/MD.0000000000050966)
Supplement: Supplementary file 4 [file medi-105-e50966-s004.docx]

| Exposure | Outcome | SNP | Effect allele | Other allele | β | Se | P | F- statistic |
| --- | --- | --- | --- | --- | --- | --- | --- | --- |
| Phosphate | AAC | rs10013062 | G | A | 0.014 | 0.003 | 3.54E-08 | 30.39 |
|  |  | rs10051765 | C | T | -0.051 | 0.003 | 3.89E-88 | 396.37 |
|  |  | rs10279171 | T | A | 0.018 | 0.003 | 9.73E-10 | 37.38 |
|  |  | rs10282449 | A | T | 0.023 | 0.003 | 1.32E-12 | 50.30 |
|  |  | rs10401230 | G | A | -0.014 | 0.002 | 1.37E-08 | 32.24 |
|  |  | rs10415758 | T | A | -0.016 | 0.002 | 6.58E-11 | 42.64 |
|  |  | rs10743976 | C | A | 0.042 | 0.003 | 3.31E-34 | 148.75 |
|  |  | rs10896012 | C | T | -0.026 | 0.003 | 3.95E-18 | 75.36 |
|  |  | rs10900829 | G | A | 0.018 | 0.002 | 2.46E-13 | 53.60 |
|  |  | rs11021221 | A | T | -0.018 | 0.003 | 2.53E-08 | 31.04 |
|  |  | rs11078597 | C | T | 0.034 | 0.003 | 5.33E-28 | 120.37 |
|  |  | rs11144134 | C | T | 0.027 | 0.004 | 5.90E-10 | 38.36 |
|  |  | rs111723017 | C | G | 0.033 | 0.004 | 9.57E-15 | 59.99 |
|  |  | rs11191643 | G | C | 0.017 | 0.002 | 2.04E-12 | 49.45 |
|  |  | rs113487566 | T | C | -0.064 | 0.008 | 3.28E-16 | 66.64 |
|  |  | rs11646780 | A | T | -0.016 | 0.002 | 8.64E-11 | 42.11 |
|  |  | rs11684211 | A | G | 0.016 | 0.003 | 2.78E-10 | 39.83 |
|  |  | rs11705850 | C | T | -0.028 | 0.005 | 9.30E-10 | 37.47 |
|  |  | rs11801716 | T | C | -0.033 | 0.006 | 1.10E-08 | 32.66 |
|  |  | rs12044944 | T | C | 0.018 | 0.003 | 6.90E-09 | 33.57 |
|  |  | rs12059422 | C | T | 0.091 | 0.008 | 1.84E-29 | 127.04 |
|  |  | rs12132412 | G | A | 0.109 | 0.003 | 1.00E-200 | 1884.99 |
|  |  | rs12409158 | C | G | -0.040 | 0.007 | 1.55E-08 | 31.99 |
|  |  | rs12518871 | T | C | -0.016 | 0.002 | 3.45E-10 | 39.41 |
|  |  | rs12710029 | C | T | -0.018 | 0.003 | 6.83E-10 | 38.07 |
|  |  | rs12727188 | G | T | -0.034 | 0.004 | 4.84E-15 | 61.33 |
|  |  | rs12769781 | A | G | 0.023 | 0.003 | 5.67E-14 | 56.49 |
|  |  | rs1286070 | C | T | -0.026 | 0.003 | 2.06E-15 | 63.02 |
|  |  | rs12902894 | C | A | 0.027 | 0.002 | 4.94E-29 | 125.08 |
|  |  | rs12941512 | T | C | -0.020 | 0.002 | 2.26E-16 | 67.37 |
|  |  | rs12985272 | A | G | 0.039 | 0.006 | 7.52E-12 | 46.89 |
|  |  | rs13208790 | C | T | 0.015 | 0.003 | 6.34E-09 | 33.73 |
|  |  | rs13232861 | G | A | 0.020 | 0.003 | 1.79E-10 | 40.68 |
|  |  | rs13273161 | T | A | 0.019 | 0.003 | 1.20E-12 | 50.48 |
|  |  | rs138893847 | GTCAT | G | -0.041 | 0.004 | 1.42E-22 | 95.59 |
|  |  | rs139796809 | T | C | -0.018 | 0.003 | 1.49E-11 | 45.55 |
|  |  | rs1412673 | G | C | 0.016 | 0.003 | 4.48E-08 | 29.93 |
|  |  | rs1432679 | T | C | 0.015 | 0.002 | 1.22E-09 | 36.94 |
|  |  | rs144214505 | T | C | -0.036 | 0.006 | 3.66E-09 | 34.80 |
|  |  | rs145330264 | C | A | -0.200 | 0.018 | 4.25E-30 | 129.96 |
|  |  | rs145978363 | G | A | -0.045 | 0.007 | 1.47E-09 | 36.58 |
|  |  | rs1468908 | G | A | 0.031 | 0.002 | 1.95E-37 | 163.55 |
|  |  | rs1483842 | C | T | -0.017 | 0.002 | 1.56E-12 | 49.98 |
|  |  | rs1570603 | A | G | -0.014 | 0.002 | 3.04E-08 | 30.68 |
|  |  | rs16869464 | G | A | -0.187 | 0.005 | 1.00E-200 | 1534.76 |
|  |  | rs17036160 | T | C | -0.025 | 0.004 | 3.44E-11 | 43.91 |
|  |  | rs17265513 | C | T | 0.027 | 0.003 | 8.37E-19 | 78.42 |
|  |  | rs17288460 | A | G | -0.021 | 0.003 | 5.91E-11 | 42.85 |
|  |  | rs17884869 | A | G | 0.050 | 0.008 | 1.97E-10 | 40.50 |
|  |  | rs1889208 | A | G | 0.020 | 0.003 | 5.41E-15 | 61.11 |
|  |  | rs200358011 | TA | T | -0.024 | 0.003 | 9.15E-13 | 51.02 |
|  |  | rs209961 | C | T | 0.023 | 0.003 | 1.14E-17 | 73.27 |
|  |  | rs2120222 | C | A | -0.057 | 0.003 | 1.19E-62 | 279.20 |
|  |  | rs2177470 | A | T | -0.017 | 0.003 | 1.69E-10 | 40.80 |
|  |  | rs2230742 | G | A | -0.025 | 0.003 | 8.49E-14 | 55.69 |
|  |  | rs2303917 | A | G | -0.023 | 0.004 | 6.21E-09 | 33.77 |
|  |  | rs2420055 | C | T | -0.022 | 0.003 | 3.39E-11 | 43.95 |
|  |  | rs2460421 | T | A | -0.013 | 0.002 | 2.87E-08 | 30.79 |
|  |  | rs273587 | T | A | -0.019 | 0.003 | 7.66E-13 | 51.37 |
|  |  | rs2797964 | T | C | 0.037 | 0.004 | 4.20E-18 | 75.23 |
|  |  | rs2909212 | G | A | 0.015 | 0.003 | 2.15E-08 | 31.36 |
|  |  | rs2941483 | G | A | 0.018 | 0.002 | 1.12E-13 | 55.15 |
|  |  | rs2955382 | T | C | -0.020 | 0.002 | 2.37E-15 | 62.74 |
|  |  | rs2959652 | T | G | -0.023 | 0.004 | 3.38E-08 | 30.48 |
|  |  | rs296847 | T | G | 0.015 | 0.003 | 9.72E-09 | 32.90 |
|  |  | rs2970818 | A | T | 0.129 | 0.004 | 1.00E-200 | 1051.13 |
|  |  | rs3020333 | G | A | -0.025 | 0.002 | 2.93E-25 | 107.85 |
|  |  | rs308032 | T | G | -0.018 | 0.003 | 5.57E-10 | 38.47 |
|  |  | rs3091842 | A | G | -0.054 | 0.006 | 1.15E-18 | 77.80 |
|  |  | rs3213473 | A | T | 0.083 | 0.007 | 1.14E-31 | 137.15 |
|  |  | rs339969 | A | C | 0.017 | 0.002 | 2.28E-11 | 44.72 |
|  |  | rs34933034 | A | G | -0.036 | 0.003 | 3.07E-26 | 112.32 |
|  |  | rs35026779 | T | C | -0.026 | 0.004 | 1.09E-10 | 41.66 |
|  |  | rs35552719 | T | G | 0.031 | 0.003 | 5.82E-29 | 124.75 |
|  |  | rs370250672 | GT | G | -0.016 | 0.003 | 1.60E-08 | 31.93 |
|  |  | rs371380082 | CA | C | 0.017 | 0.003 | 9.84E-09 | 32.88 |
|  |  | rs375640583 | CTT | C | -0.017 | 0.003 | 1.28E-09 | 36.84 |
|  |  | rs3813498 | T | C | 0.032 | 0.003 | 1.98E-24 | 104.06 |
|  |  | rs3897629 | A | T | -0.015 | 0.002 | 8.01E-10 | 37.76 |
|  |  | rs4072537 | C | T | -0.014 | 0.003 | 3.70E-08 | 30.30 |
|  |  | rs4077450 | T | G | -0.026 | 0.003 | 1.60E-15 | 63.52 |
|  |  | rs41278174 | A | G | 0.107 | 0.007 | 1.21E-46 | 205.72 |
|  |  | rs4410790 | C | T | 0.017 | 0.003 | 1.47E-11 | 45.58 |
|  |  | rs4509682 | A | G | -0.020 | 0.002 | 4.13E-15 | 61.65 |
|  |  | rs453639 | A | C | -0.067 | 0.003 | 4.36E-151 | 686.18 |
|  |  | rs4690098 | T | C | -0.016 | 0.003 | 1.76E-08 | 31.74 |
|  |  | rs4706554 | A | G | 0.018 | 0.002 | 3.51E-13 | 52.91 |
|  |  | rs4709746 | T | C | 0.022 | 0.004 | 1.64E-09 | 36.36 |
|  |  | rs4759844 | G | A | 0.024 | 0.003 | 3.00E-21 | 89.56 |
|  |  | rs4795607 | T | C | 0.015 | 0.003 | 1.07E-09 | 37.20 |
|  |  | rs4820324 | C | G | 0.019 | 0.002 | 4.05E-14 | 57.15 |
|  |  | rs485411 | C | T | 0.021 | 0.003 | 2.27E-13 | 53.76 |
|  |  | rs4855880 | A | G | -0.014 | 0.002 | 4.70E-08 | 29.84 |
|  |  | rs4869745 | T | C | 0.019 | 0.003 | 6.66E-13 | 51.65 |
|  |  | rs4938642 | C | G | 0.026 | 0.005 | 3.96E-08 | 30.17 |
|  |  | rs4982711 | G | T | 0.014 | 0.002 | 1.21E-08 | 32.47 |
|  |  | rs5435 | C | T | -0.014 | 0.003 | 3.01E-08 | 30.70 |
|  |  | rs55923451 | A | G | -0.021 | 0.003 | 7.00E-11 | 42.52 |
|  |  | rs55938136 | G | A | -0.027 | 0.003 | 8.59E-21 | 87.47 |
|  |  | rs56078331 | T | C | 0.053 | 0.005 | 2.04E-22 | 94.87 |
|  |  | rs56328339 | T | C | 0.032 | 0.003 | 1.15E-22 | 96.01 |
|  |  | rs56738967 | C | G | -0.032 | 0.003 | 4.02E-35 | 152.94 |
|  |  | rs56795888 | GT | G | -0.014 | 0.002 | 1.29E-08 | 32.35 |
|  |  | rs58117425 | A | G | -0.016 | 0.003 | 3.10E-08 | 30.65 |
|  |  | rs58468752 | TA | T | -0.016 | 0.002 | 8.88E-11 | 42.06 |
|  |  | rs596940 | C | T | 0.032 | 0.005 | 7.11E-12 | 47.00 |
|  |  | rs6136492 | T | C | -0.017 | 0.003 | 7.73E-09 | 33.34 |
|  |  | rs61870274 | A | C | 0.036 | 0.005 | 1.14E-15 | 64.18 |
|  |  | rs61909254 | C | T | 0.063 | 0.004 | 7.81E-72 | 321.40 |
|  |  | rs62289280 | C | T | -0.018 | 0.003 | 5.51E-12 | 47.50 |
|  |  | rs62383568 | C | T | -0.015 | 0.003 | 4.53E-09 | 34.38 |
|  |  | rs653178 | T | C | -0.025 | 0.002 | 2.60E-24 | 103.52 |
|  |  | rs6650130 | T | A | 0.020 | 0.003 | 1.59E-13 | 54.46 |
|  |  | rs6751217 | T | C | 0.018 | 0.003 | 4.51E-13 | 52.41 |
|  |  | rs6780593 | A | G | -0.028 | 0.003 | 5.29E-17 | 70.24 |
|  |  | rs6841258 | T | C | 0.019 | 0.003 | 4.15E-09 | 34.55 |
|  |  | rs6911602 | A | G | 0.051 | 0.004 | 2.81E-46 | 204.07 |
|  |  | rs6983239 | T | G | 0.024 | 0.003 | 4.60E-16 | 65.97 |
|  |  | rs7017252 | T | C | 0.014 | 0.003 | 4.47E-08 | 29.94 |
|  |  | rs7033278 | T | C | 0.022 | 0.003 | 5.43E-16 | 65.65 |
|  |  | rs706300 | G | A | -0.034 | 0.003 | 1.25E-22 | 95.84 |
|  |  | rs71368113 | G | C | -0.028 | 0.005 | 4.62E-08 | 29.87 |
|  |  | rs71413981 | A | G | -0.019 | 0.003 | 4.90E-09 | 34.23 |
|  |  | rs71641333 | A | T | -0.028 | 0.005 | 3.87E-09 | 34.69 |
|  |  | rs72660359 | T | G | 0.092 | 0.007 | 8.83E-46 | 201.78 |
|  |  | rs72716022 | T | C | -0.021 | 0.003 | 9.30E-11 | 41.97 |
|  |  | rs72974112 | T | C | -0.018 | 0.003 | 4.80E-10 | 38.76 |
|  |  | rs73186030 | T | C | -0.073 | 0.004 | 1.86E-88 | 397.82 |
|  |  | rs7324259 | T | G | 0.023 | 0.004 | 2.99E-10 | 39.68 |
|  |  | rs73632745 | T | C | 0.026 | 0.005 | 3.87E-08 | 30.22 |
|  |  | rs74551305 | C | T | 0.046 | 0.006 | 8.82E-17 | 69.22 |
|  |  | rs74737968 | G | C | -0.035 | 0.004 | 3.41E-18 | 75.65 |
|  |  | rs7529925 | T | C | 0.019 | 0.003 | 4.42E-12 | 47.93 |
|  |  | rs7555660 | T | C | 0.057 | 0.004 | 8.10E-39 | 169.87 |
|  |  | rs7625643 | G | A | 0.016 | 0.002 | 9.20E-11 | 41.99 |
|  |  | rs763704 | C | T | 0.019 | 0.003 | 4.83E-14 | 56.80 |
|  |  | rs76527351 | G | A | -0.027 | 0.003 | 9.73E-21 | 87.23 |
|  |  | rs7672820 | C | G | 0.027 | 0.003 | 6.67E-18 | 74.32 |
|  |  | rs7705189 | G | A | -0.015 | 0.002 | 3.15E-10 | 39.58 |
|  |  | rs77149268 | T | C | 0.041 | 0.004 | 4.72E-25 | 106.90 |
|  |  | rs7726961 | T | C | -0.014 | 0.002 | 4.34E-09 | 34.47 |
|  |  | rs77282753 | C | T | -0.043 | 0.006 | 4.79E-14 | 56.82 |
|  |  | rs78030362 | G | A | 0.039 | 0.005 | 6.45E-17 | 69.84 |
|  |  | rs7860558 | C | T | -0.018 | 0.003 | 1.93E-11 | 45.04 |
|  |  | rs79472736 | A | G | -0.044 | 0.007 | 3.11E-11 | 44.11 |
|  |  | rs8072297 | T | A | -0.036 | 0.003 | 1.66E-33 | 145.55 |
|  |  | rs8075449 | T | C | -0.017 | 0.003 | 3.99E-10 | 39.12 |
|  |  | rs8105161 | C | T | -0.022 | 0.003 | 1.06E-10 | 41.71 |
|  |  | rs838717 | A | G | 0.031 | 0.002 | 1.34E-35 | 155.12 |
|  |  | rs872386 | A | G | -0.024 | 0.002 | 2.92E-23 | 98.73 |
|  |  | rs878919 | C | T | 0.020 | 0.002 | 1.10E-15 | 64.25 |
|  |  | rs896788 | T | C | 0.027 | 0.003 | 2.77E-15 | 62.43 |
|  |  | rs912981 | C | A | -0.016 | 0.002 | 2.28E-11 | 44.71 |
|  |  | rs9388490 | T | C | 0.017 | 0.002 | 4.46E-12 | 47.92 |
|  |  | rs9574586 | C | T | -0.015 | 0.003 | 1.36E-08 | 32.25 |
|  |  | rs9745989 | C | T | 0.016 | 0.003 | 1.15E-09 | 37.06 |
|  |  | rs9799552 | A | C | 0.022 | 0.003 | 1.15E-13 | 55.10 |
